# Microbial biofilms on macroalgae harbour diverse integron gene cassettes

**DOI:** 10.1099/mic.0.001446

**Published:** 2024-03-15

**Authors:** Stefano Freddi, Vaheesan Rajabal, Sasha G. Tetu, Michael R. Gillings, Anahit Penesyan

**Affiliations:** 1 School of Natural Sciences, Faculty of Science and Engineering, Macquarie University, NSW 2109, Australia; 2 Australian Research Council Centre of Excellence in Synthetic Biology, Macquarie University, NSW 2109, Australia

**Keywords:** holobiont, microbiome, pangenome, epiphytic microbial communities, microbial interactions, microbial adaptation

## Abstract

Integrons are genetic platforms that capture, rearrange and express mobile modules called gene cassettes. The best characterized gene cassettes encode antibiotic resistance, but the function of most integron gene cassettes remains unknown. Functional predictions suggest that many gene cassettes could encode proteins that facilitate interactions with other cells and with the extracellular environment. Because cell interactions are essential for biofilm stability, we sequenced gene cassettes from biofilms growing on the surface of the marine macroalgae *Ulva australis* and *Sargassum linearifolium*. Algal samples were obtained from coastal rock platforms around Sydney, Australia, using seawater as a control. We demonstrated that integrons in microbial biofilms did not sample genes randomly from the surrounding seawater, but harboured specific functions that potentially provided an adaptive advantage to both the bacterial cells in biofilm communities and their macroalgal host. Further, integron gene cassettes had a well-defined spatial distribution, suggesting that each bacterial biofilm acquired these genetic elements via sampling from a large but localized pool of gene cassettes. These findings suggest two forms of filtering: a selective acquisition of different integron-containing bacterial species into the distinct biofilms on *Ulva* and *Sargassum* surfaces, and a selective retention of unique populations of gene cassettes at each sampling location.

## Introduction

Integrons are genetic platforms that can capture, express, excise and rearrange gene-carrying elements called gene cassettes [1]. Gene cassettes are compact elements which generally consist of a single ORF alongside a recombination site, termed *attC* [2], and can occur as circular elements or integrated into bacterial chromosomes or mobile DNAs. Integrons share a common structure. They typically contain an integron-integrase gene (*IntI*), which encodes a tyrosine recombinase. This facilitates the insertion of circular gene cassettes by mediating site-recombination between the attachment site of the integron (*attI*) and the gene cassette *attC* site [3].

Integrons can be mobile or sedentary. Mobile integrons are embedded in insertion sequences, plasmids or transposons, often carry antibiotic resistance genes, and can easily be transferred to other cells via horizontal gene transfer (HGT). In contrast, sedentary integrons are found on bacterial chromosomes, are spread via vertical transmission and can encode hundreds of gene cassettes of mostly unknown function [1]. Given the clinical relevance of mobile integrons, most studies have focused on their role in capturing and spreading gene cassettes that confer antibiotic resistance. However, integrons have access to a much broader and diverse pool of gene cassettes in the environment. Previous work indicates any 0.3 g soil sample can contain between 4000 and 18 000 unique gene cassettes, and the gene cassette population exhibits spatial turnover at distances of <100 m [4]. Consequently, gene cassettes represent a repository of enormous functional and genetic diversity.

Gene cassettes sampled outside of clinical settings are predicted to encode proteins that perform a wide variety of functions. These include toxin–antitoxin systems, DNA modification, phage-related functions and acetyltransferases. In addition, estimates suggest that a third of gene cassettes encode proteins that contain a signal peptide associated with cellular export or transmembrane domains [1], indicating a possible membrane or extracellular localization. This suggests a role in mediating interactions with other cells and the environment. Since cell interactions are a fundamental aspect of biofilms, it has therefore been hypothesized that gene cassettes might be involved in cell–cell interactions that are critical for the biofilm mode of life [1].

Marine macroalgae harbour microbial biofilms which can provide mutual benefits to both microbes and the host. The surfaces of marine macroalgae provide a favourable habitat for bacterial colonization and proliferation due to the production and release of organic nutrients [5,6]. In return, bacteria can supply their host with nutrients that are essential for macroalgal growth [7] and prevent the settlement of unwanted organisms on algal surfaces [6,8, 9]. This suggests that bacteria in macroalgal-associated biofilms exhibit complex interactions not only between microbial cells, but also between microbes and their eukaryotic host.

Biofilms are of particular interest for the study of HGT and here we look at one element contributing to HGT in biofilm communities: integron gene cassettes. The objective of this study was to assess the abundance and distribution of integron gene cassettes in macroalgal-associated biofilms and in the surrounding seawater. Furthermore, we explored the functional traits carried by integron gene cassettes to investigate their potential role in facilitating interactions that could benefit bacterial cells and the host.

## Methods

### Sample collection and preparation

**Fig. 1.**
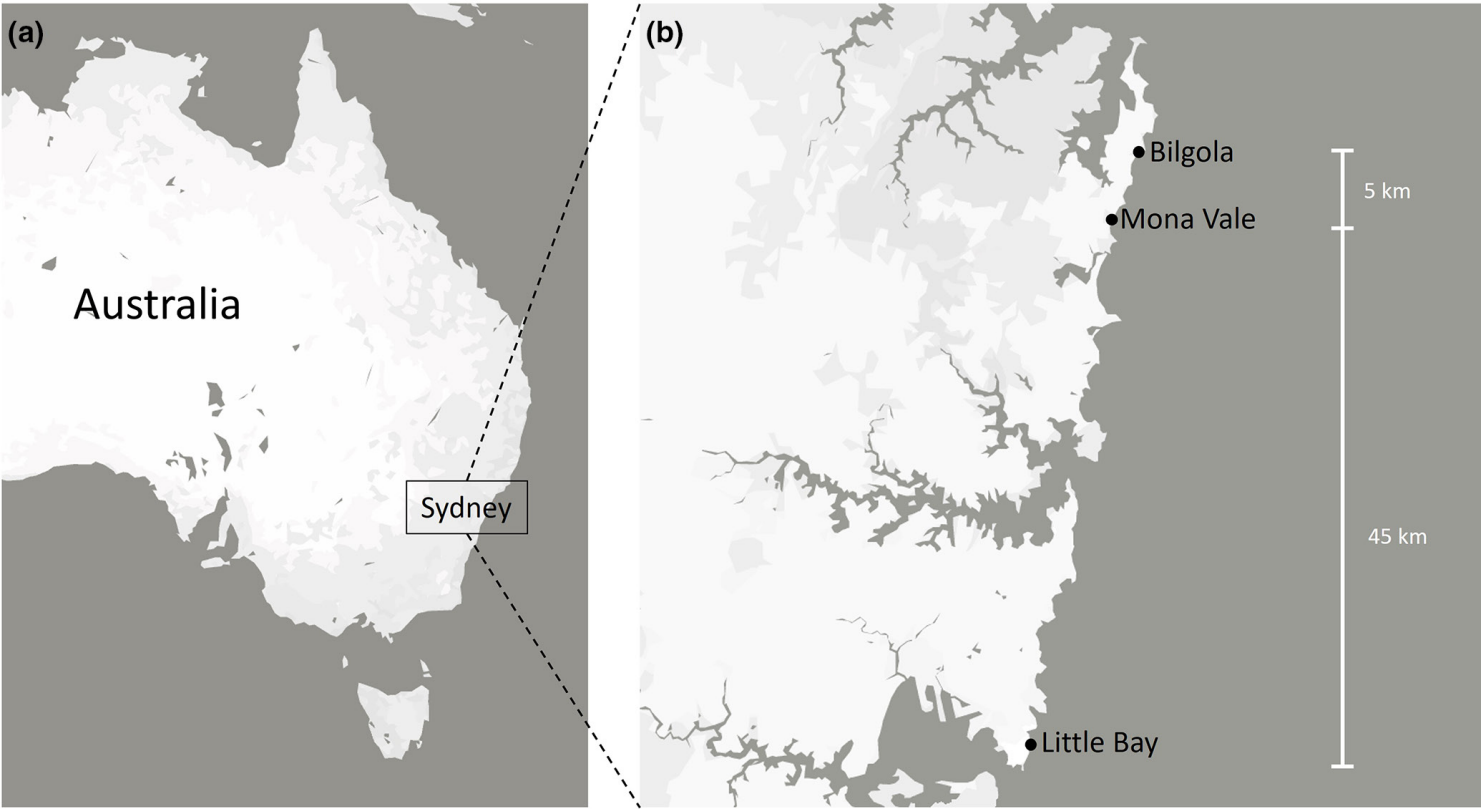
(a) Map of the locations from which marine macroalgae and seawater samples were collected . (b) Distances between the sampling sites.

Thalli of *Ulva australis* and *Sargassum linearifolium* were sampled on 11 March 2022 from the rock platforms of Little Bay (33.9802° S 151.2525° E), Mona Vale (33.6784° S 151.3167° E) and Bilgola (33.6476° S, 151.3276° E), on the coastline of Sydney, Australia (Fig. 1). Three samples of each macroalgal species were collected from three separate rock pools at each of the three sampling locations, placed in zip lock bags and stored on ice. Seawater (2 litres) was also sampled from the same rock pools and stored in a cooler.

All samples were collected on the same day, and immediately transferred to the laboratory for processing. Each macroalgal sample was washed with 50 ml of sterile isotonic solution to remove debris and loosely attached bacteria, and then sealed in individual bags with a volume of 50 ml PBS solution containing 0.01 % Tween 20 (PBST). The bagged and sealed samples were sonicated for 10 min at 40 kHz and then placed in a shaker for 30 min at 225 r.p.m. to dislodge bacterial cells from the surface of the macroalgae. The resulting suspension was filtered using a 40 µm filter to remove algal debris, centrifuged at 7830 r.p.m. for 10 min, and then resuspended in 1.5 ml PBS (0.01 M, pH 7.4) The 2 litre seawater samples were filtered sequentially using 40 and 0.2 µm pore size filters. Cells from the 0.2 µm filters were collected by suspending the filters in 1.5 ml PBST and vortexing. The resulting suspension was then centrifuged using the same parameters described above.

### DNA extraction and PCR amplification

DNA was extracted using the bead-beating method described by Gillings [10]. Briefly, cells were resuspended in 780 µl PBS with 122 µl MT buffer (MP Biomedicals) and then processed using the FastPrep machine for two cycles of 40 s at 5 m s^–1^. The samples were centrifuged at 14 000 r.p.m. for 5 min and the supernatant (~600 µl) was mixed with 150 µl of protein precipitation solution and vortexed. Another centrifugation cycle was performed as above, and 700 µl of binding matrix (MP Biomedicals) was added to the recovered supernatant (~700 µl). To increase the efficiency of DNA binding the samples were placed on a rotary shaker for 30 min and then centrifuged for 2 min at 14 000 r.p.m. The supernatant was discarded, and the resulting pellet was allowed to dry before being resuspended in 200 µl TE buffer. A final centrifugation was performed at 14 000 r.p.m. for 3 min after which 160 µl of supernatant was recovered, placed into a fresh tube and stored at −20 °C. The DNA concentration of all samples was measured using both Qubit and Nanodrop technology (Thermo Fisher Scientific).

The extracted DNA was used as a template for PCR amplification of integron gene cassettes using Phusion DNA Polymerase (NEB). Gene cassettes were amplified using the primers HS287 (5′-GCSGCTKANCTCVRRCGTTAGSC-3′) and HS286 (5′-TCSGCTKGARCGAMTTGTTAGVC-3′) which target conserved sequences of integron *attC* recombination sites [11]. Using ~30 ng of DNA as a template, gene cassettes were amplified using a thermal cycler (Eppendorf Mastercycler EP Gradient S) set at 98 °C for 3 min followed by 35 cycles of 98 °C for 10 s, 60 °C for 30 s, 72 °C for 210 s and a final extension at 72 °C for 10 s. Gene cassette PCRs were performed in technical triplicates for each sample. PCR products were assessed using 1.5 % agarose gel electrophoresis.

### DNA library preparation and sequencing

After observing a banding pattern in PCR products for all 27 samples, representing three biological replicates for each macroalgal species and seawater samples, the PCR-amplified samples were sequenced using ONT (Oxford Nanopore Technologies) long-read sequencing [12]. Briefly, PCR products obtained from technical triplicates were pooled and cleaned using AMPure XP beads (Beckman Coulter) as per the manufacturer’s protocol. Cleaned products from each individual sample were then barcoded and equimolar concentrations of each sample were multiplexed into two separate libraries using the ONT Native Barcoding Expansion Kit (EXP-NBD104 and EXP-NBD114) and the ONT Ligation Sequencing Kit (SQK-LSK 110). Sequencing was carried out in a MinION MK 1B sequencing device with R9.4 flow cell. Sequencing was allowed to run between 15 and 23 h until approximately 12 million reads were generated in total. Base-calling was performed with Guppy v6.3.2 with default parameters using the high-accuracy base-calling model.

### Sequence processing of integron gene cassettes

ONT sequencing reads were processed as previously described [13]. Briefly, gene cassette sequences from the HS287/HS286 PCR products were initially filtered based on average quality scores. This was performed using NanoFilt v2.8 (parameters: -q 10), which removes reads with an average q score <10. To account for amplicon reads overlapping with larger potential templates, an assembly of these reads into larger cassette arrays was carried out on the Gadi supercomputer (part of Australia’s National Computing Infrastructure) using Canu v2.2 in grid mode (parameters: genomeSize=100 k, minReadLength=250, minOverlapLength=200, corMinCoverage=0, corOutC9verage=20 000, corMhapSensitivity=high, maxInputCoverage=20 000, purgeOverlaps=aggressive). The extraction of assembled contigs and unassembled reads was done with the tgStoreDump script within Canu (parameters: -consensus –fasta). This was followed by the removal of any redundancies using the dedupe.sh script which is available from the BBTools package v38.96 with default parameters. Finally, all consensus sequences were corrected with four rounds of polishing using Racon v1.5.0.

### Abundance and richness of integron gene cassettes

Gene cassette ORFs and their translated protein sequences were identified using Prodigal v2.6.3 [14] in metagenome mode (parameters: -p meta). Unique gene cassette ORFs were obtained using CD-HIT v4.8.1 (sequence identity threshold=0.97, alignment coverage=0.9). The gene cassette reads from each sample were mapped against the unique gene cassettes, and their relative abundance was calculated as mean depth per million bases. The number of unique and shared gene cassette ORFs found in each sample was visualized with Venn diagrams. Samples were grouped and compared based on sample type (derived from a particular alga or seawater) across all locations, as well as by sample type within locations, and by same sample type across different locations. These groups were processed with CD-HIT v4.8.1 using the same parameters described above and then compared with each other to determine the abundance of shared and unique gene cassette ORFs.

### Functional characterization of gene cassettes

The functional diversity of gene cassettes was assessed using both SeqScreen [15], which assigns functional annotations and putative taxonomic origin for gene cassettes (with an e-value cut-off <0.01), and the online Batch Web CD-Search Tool from the National Center for Biotechnology Information (NCBI, with an e-value cut-off <0.01), which assigns genes to putative functional categories, based on searches against the Clusters of Orthologous Groups (COGs) database. In addition, to explore the possible cellular localization of gene products, all cassette genes were screened for the presence of signal peptide cleavage sites using SignalP v5.0 [16] with default parameters. Using these parameters SignalP searches for all prokaryotic signal peptide tag sequences, which target proteins into, or across, membranes.

### Predicting the taxonomic origin of gene cassettes

The taxonomic origin of gene cassettes was predicted using the attC-taxa.sh script (available at: https://github.com/timghaly/integron-filtering) developed by Ghaly *et al*. [17] with default parameters. The script compares *attC* sites in sequence data with the conserved sequences of *attC* sites from the chromosomal integrons of 11 bacterial taxa known to contain distinct *attC* genes, and allows the identification of the possible source taxon.

### Data visualization and statistical analyses

Cluster heatmaps were created using ampvis2 (v2.7.31) while custom figures were produced with ggplot2 (v3.3.6). Venn diagrams were made using the Multiple List Comparator (http://www.molbiotools.com/listcompare.html). The abundances and distributions of taxonomic groups and integron gene cassettes between sample types and locations were assessed, compared and analysed using beta diversity analyses, Bray–Curtis dissimilarity matrices, relative abundances, principal coordinate analysis (PCoA) and permutational multivariate analysis of variance (PERMANOVA).

## Results and discussion

### Abundance and distribution of gene cassettes

A total of 80 601 gene cassette arrays were recovered from the 27 samples analysed in this study (μ=2985; max=4447; min=1173). These arrays ranged from 500 to 12 067 bp in length, with 92.2 % of assembled arrays falling between 800 and 2000 bp. Cassette arrays contained a total of 220 158 ORFs (μ=8107; min=2316; max=13 861) with 99 724 unique gene cassette ORF sequences. These varied in size from 60 to 2931 bp in length, with 90.3 % of sequences falling in the range 150–800 bp.

**Fig. 2.**
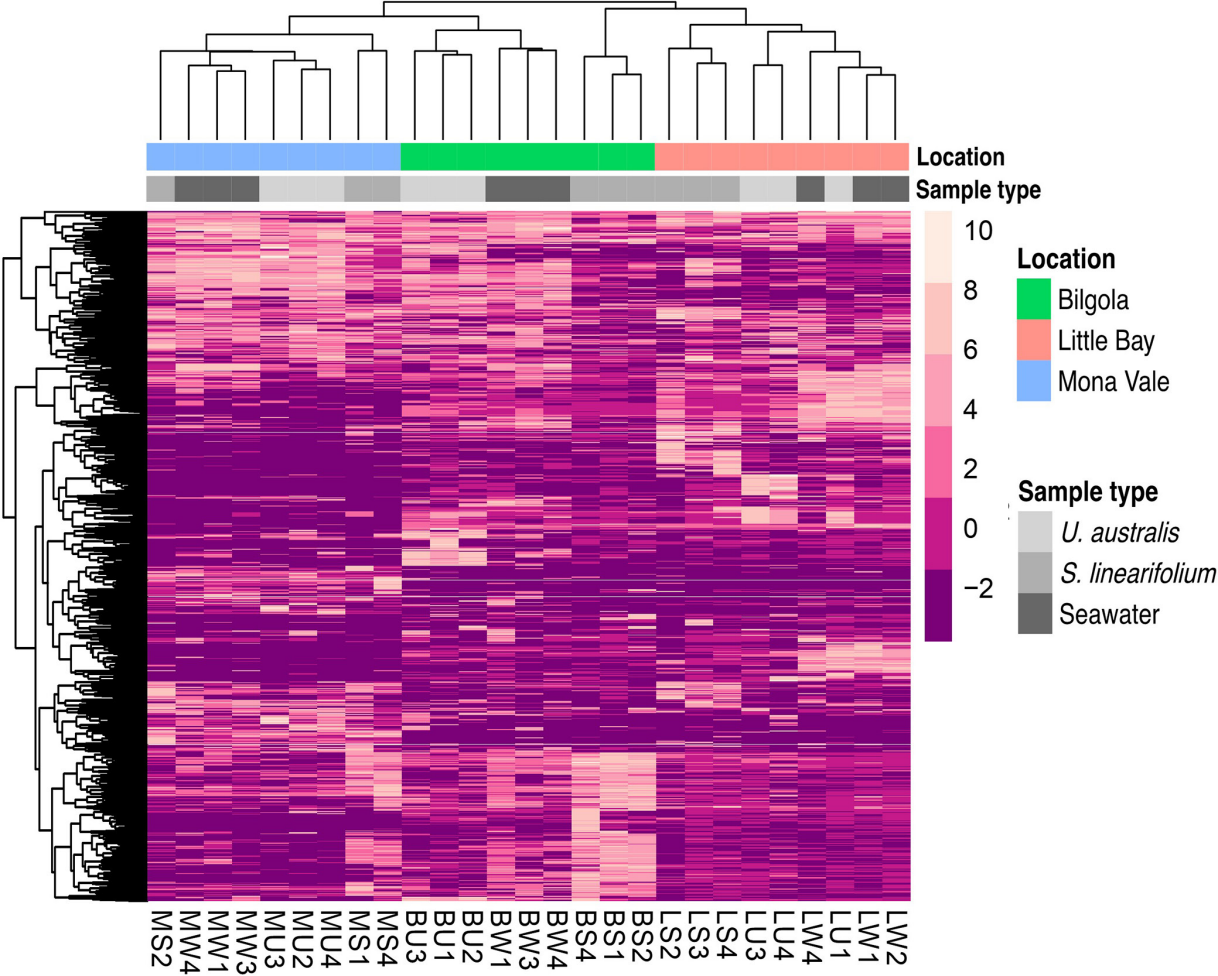
The abundance and distribution of the top 1000 most abundant integron gene cassette ORFs among the samples. The heatmap shows a clear clustering based on location. Data were transformed using the centre log ratio transformation, and differences in abundances between samples are represented by the scalebar (shades of purple). Dendrograms show similarities between rows and columns, which were ordered according to hierarchical clustering. The top bar shows locations from which samples were obtained and correspond to Bilgola (green), Little Bay (orange) and Mona Vale (blue); the bottom bar indicates the sample type (shown in shades of grey).

The relative abundances of gene cassette ORFs present in all samples were used to generate a heatmap (Fig. 2) to reveal distribution patterns of integron gene cassette ORFs among samples. Gene cassettes primarily clustered based on the sample location rather than by sample type (Fig. 2). These findings could indicate that each geographical location has a distinct pool of gene cassettes that bacteria can sample, further supported by PERMANOVA (Table 1).

**Table 1.** Output of the PERMANOVA performed on the integron gene cassette data comparing sample types and locations Values with asterisks indicate statistically significant differences between samples. The last two columns represent *F*-values, and *P*-values associated with the *F* statistic.

	Sample type		
**Sample1**	**Sample 2**	***F***	***Pr*** **(>** ***F*** **)**
*S. linearifolium*	*U. australis*	1.961	0.001*
*U. australis*	Seawater	1.662	0.011*
Seawater	*S. linearifolium*	2.264	0.001*
	**Location**		
**Site 1**	**Site 2**	***F***	***Pr*** **(>** ***F*** **)**
Bilgola	Mona Vale	1.602	0.013*
Mona Vale	Little Bay	2.21	0.002*
Little Bay	Bilgola	2.312	0.001*

Such spatial distribution of gene cassettes could be explained by considering two main factors: dispersal and selection. Integron gene cassettes are mobile elements and, therefore, can be directly involved in HGT events. This is particularly evident for gene cassettes resident within mobile integrons, often carrying antibiotic resistance genes (ARGs), which have spread to every habitat on Earth [4,18]. However, integron gene cassettes resident within sedentary chromosomal locations, where the occurrence of HGT events is rare, are probably subject to vertical transmission and thus may stay largely localized within a short distance from their origin [19].

The spatial distribution patterns observed in this study align with these principles. Our findings revealed a distinct spatial arrangement of gene cassettes (Fig. 2). Notably, the clustering of samples based on geographical location, rather than sample type, underscores the significant influence of local environmental factors on the distribution of gene cassettes. Moreover, the observation that samples from *S. linearifolium* in Bilgola exhibited closer relatedness to those from Little Bay, despite the greater geographical separation from Mona Vale, highlights the complex interplay between dispersal mechanisms and local selection pressures, as well as specific characteristics of the host.

Further analysis utilizing PERMANOVA confirmed significant differences in the distribution of gene cassettes between sample types and across sampling locations (Table 1), emphasizing the multifaceted nature of the processes driving their spatial distribution.

**Fig. 3.**
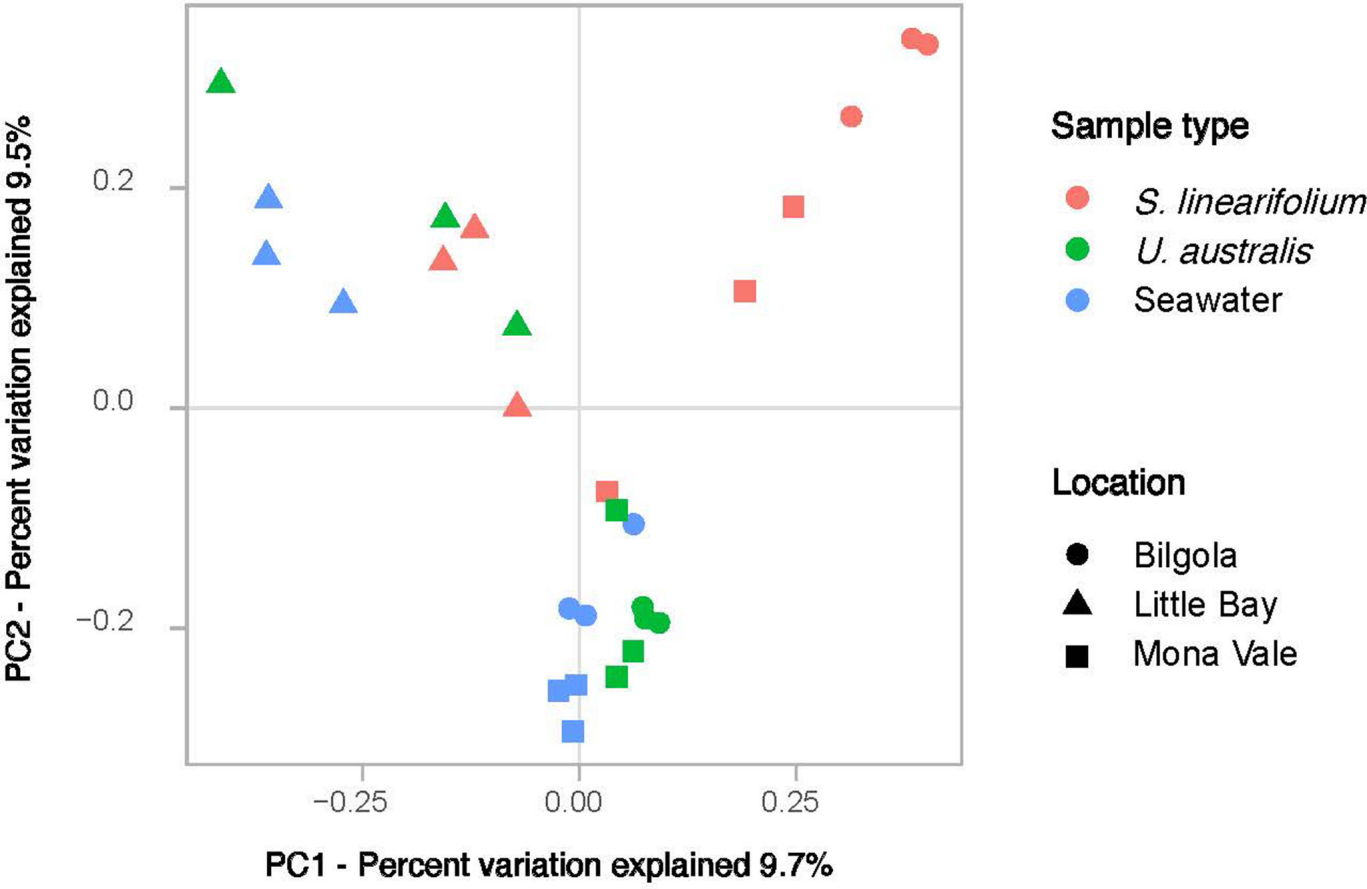
Principal coordinate analysis plot indicating compositional differences in the gene cassette suites from each of the 27 samples, generated using the Bray–Curtis dissimilarity metric. Data points are colour-coded based on the sample type and shaped according to the location from which they were sampled.

While PERMANOVA showed that location was a significant driver of gene cassette composition in both biofilm and seawater samples, PCoA indicated that gene cassette profiles were less distinct between the Mona Vale and Bilgola samples compared to those from Little Bay, which clustered together separately from the other locations (Fig. 3). This could be explained by the relatively short distance between sampling locations, since Mona Vale and Bilgola are about 5 km apart, as opposed to Little Bay, which is 45 km from these sites (Fig. 1). It is likely that, given their proximity, there is some level of gene exchange between Mona Vale and Bilgola. Moreover, due to close proximity, these two locations are likely to be exposed to similar selective pressures that may ultimately drive the recruitment of similar gene cassettes on the surfaces of macroalgae from the available pool. It is worth noting that analyses based just on the 1000 most abundant cassettes (Fig. 2) do not show the same pattern, indicating that distribution of highly abundant cassettes may be less shaped by site proximity.

Previous studies on gene cassettes recovered from terrestrial environments suggested short spatial scales for gene cassette dispersal in soil, as totally new sets of thousands of gene cassettes could be found between samples taken only 100 m apart [4]. However, given the inherent nature of aquatic systems, it is expected that gene cassettes in aquatic environments could be transported and dispersed across larger distances through water, unlike terrestrial environments where gene cassettes that arise from a single cell could remain localized on a microscale. The distribution of gene cassettes in aquatic environments would then depend on the ability of their associated phenotypic traits to confer adaptation to the selective pressures present at each location.

**Fig. 4.**
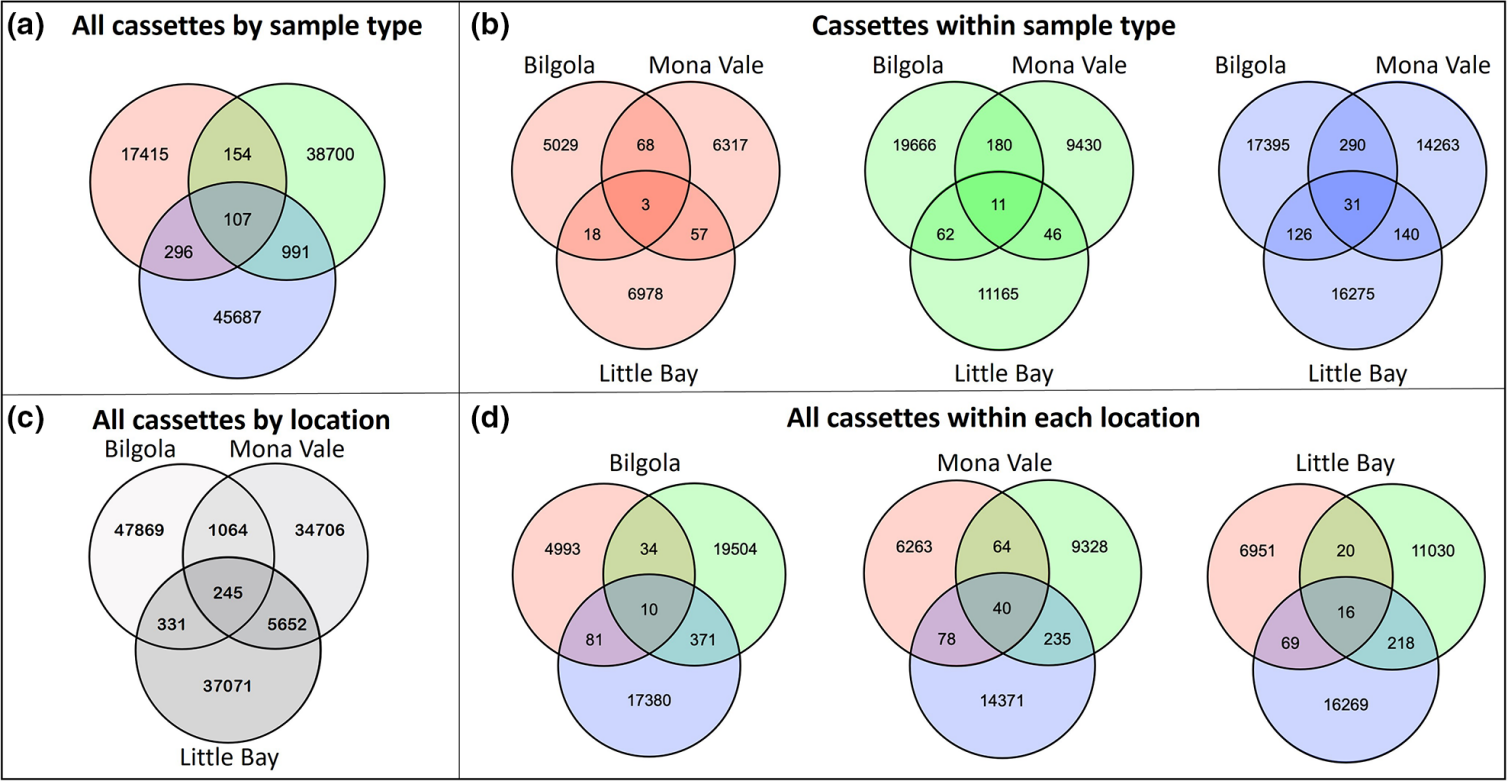
Venn diagrams showing the number of unique integron gene cassette ORFs and those shared between the microbial communities associated with *U. australis* (green), *S. linearifolium* (orange) and seawater (blue), and in different locations. Samples were combined based on sample type (**a, b**) and location (c, d; samples from different locations are shown in shades of grey in c).

The number of gene cassettes shared between the microbial communities of *S. linearifolium*, *U. australis* and seawater were minimal, considering that thousands of unique gene cassettes were recovered for each sample type (Fig. 4a). Comparisons were also made by grouping gene cassettes shared between microbial communities present in the same sample type (Fig. 4c) and at different locations (Fig. 4b). Samples obtained from *S. linearifolium*, *U. australis* and seawater, even from the same location (Fig. 4b), were still characterized by thousands of unique gene cassettes, indicating that the pool of gene cassettes recruited by microbial communities on each type of macroalga were considerably different from each other and from those present in the surrounding seawater. Likewise, the small number of shared gene cassettes observed from the same sample type from different locations (Fig. 4c) emphasizes the importance of geographical location for the recruitment of gene cassettes from the pool of available elements.

Ultimately, macroalga must obtain their microbial partners from the local environment. The lack of commonality between the gene cassettes found in different sample types and at different locations has implications for assembly of these microbial biofilms and their gene cassette cargo. Either the micro-organisms are selected from seawater, and then enriched on the surface of the macroalga, along with their constituent gene cassettes, or there is a more direct form of horizontal transmission of these cassette elements into bacteria already residing on the macroalga. In either case, it suggests strong selection on both bacterial species and gene cassettes during biofilm formation, although this study is not able to determine the contribution of each process.

### Predicted cellular localization of gene cassette products

Previous studies have shown that a substantial proportion of the gene cassette pool encodes proteins that are either associated with the cell membrane or are exported from the cell [20,21]. These proteins can potentially facilitate interactions between cells and between cells and their external environment. To test this hypothesis, gene cassette sequences were screened for signal peptide regions, which determine whether proteins are directed towards the membrane or out of the cell. On average, 11 % of gene cassette ORFs recovered from macroalgal-associated biofilms, and 14 % of gene cassettes from seawater samples, encoded signal peptide domains. This was similar to the proportion of signal peptide sequences (13.6 %) found by Ghaly *et al*. [4] in gene cassettes recovered from soil samples collected in Antarctica and Australia. Previous work has shown gene cassette ORFs are substantially enriched in signal peptide sequences. For example, it was demonstrated that ORFs within gene cassettes contained significantly higher proportions of such sequences than seen in their associated metagenomes (Ghaly *et al*., in press).

Since integron gene cassettes can be both acquired or lost over relatively short time periods, this component of the bacterial accessory gene pool may be particularly important in conferring a competitive advantage in changing environments. Integron genes could therefore represent a source of ‘plug-in functions’, facilitating rapid adaptation of bacteria to altered conditions due to interactions with other cells or changes in the environment.

### Functional characterization of gene cassettes

**Fig. 5.**
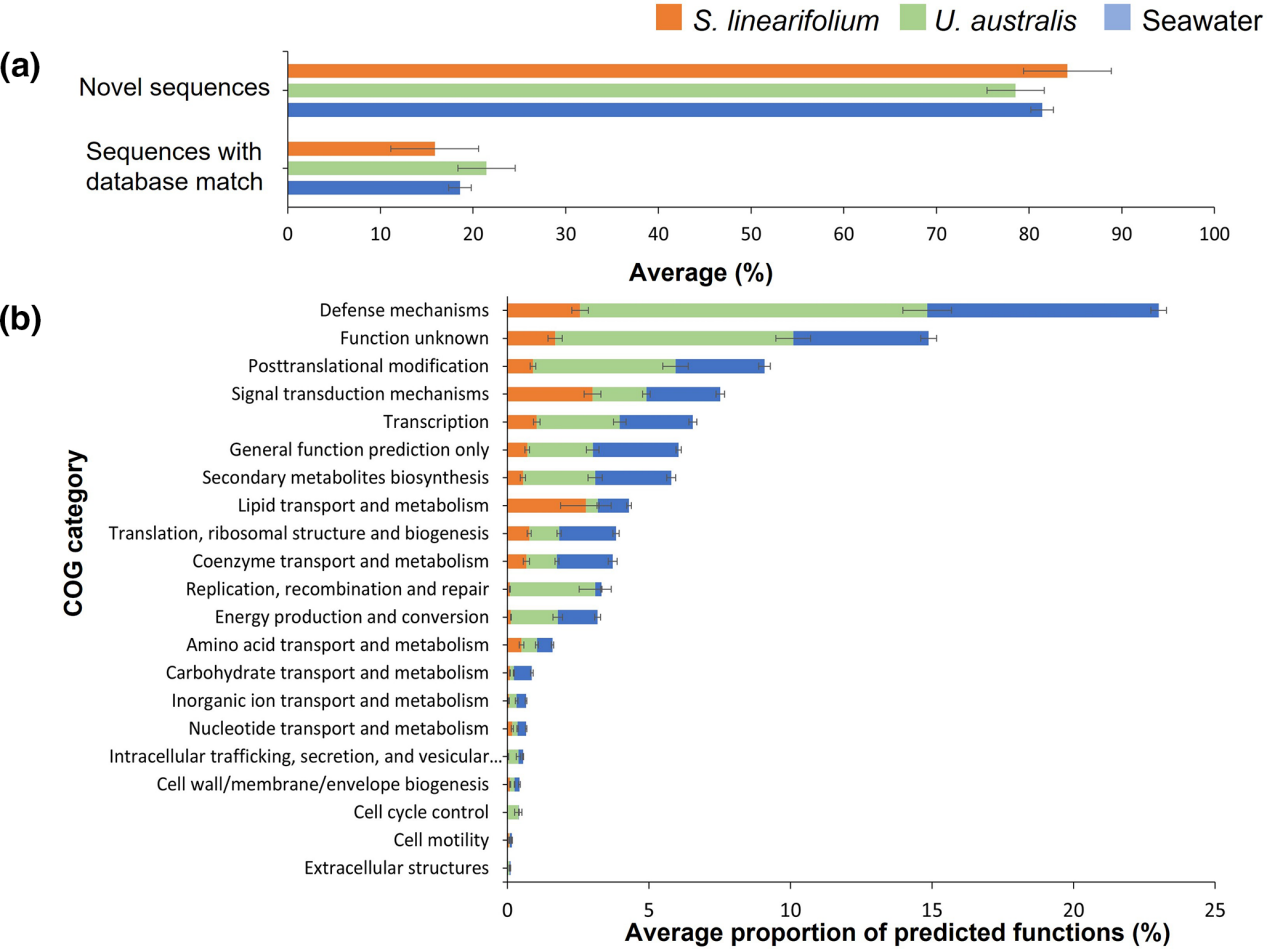
Functional analysis of integron gene cassettes recovered using the primer pair HS287/HS286. (**a**) Average percentage (±sd) of gene cassette ORFs per sample (*n*=27) encoding proteins with or without a database match. (**b**) Average (±sd) proportion of the functional categories assigned to predicted functions.

Approximately 80 % of integron gene cassette ORF sequences did not have a match in the NCBI database with an e-value cut-off <0.01 (Fig. 5a) and probably represent novel proteins. This is consistent with previous functional analyses of gene cassettes performed on a variety of terrestrial, freshwater and marine samples [13,20, 22], indicating that integron gene cassettes are an incredibly vast and untapped resource of novel functions.

On average, only ~20 % of the proteins encoded by gene cassette ORFs were assigned a COG functional category (Fig. 5a), out of which~15 % had homologues with no identified function and, therefore, were labelled under the category ‘Function unknown’ (Fig. 5b).

Among predicted functions, the most predominant COG category was ‘Defence mechanisms’ which averaged ~23 % of all predicted functions. This category was the most common for gene cassettes associated with *U. australis* (~13 %) and seawater (~9 %) samples, but not for *S. linearifolium* (~2 %) samples (Fig. 5b). Interestingly, *S. linearifolium*, like many other brown algae species, can synthesize polyphenolic compounds that are thought to provide a chemical defence against grazers and other external stressors [23]. In contrast, *U. australis* is not known to produce such bioactive compounds on its own [24] and is believed to rely mainly on its epiphytic microbial community for protection against pathogenic bacteria and biofouling organisms [25]. Our results suggest that the enrichment of integron gene cassettes involved in defence mechanisms may play a role in defining symbiotic relationships between the biofilm microbial community and *U. australis*, with biofilm bacteria contributing to the defence of not only the microbial community itself, but also the host, a type of symbiotic relationship that has been previously suggested to exist between *U. australis* and its epiphytic bacteria [26]. In contrast, *S. linearifolium* could largely rely on its own defence mechanisms in protecting itself against unwanted colonization, thus reducing the need for epibionts to perform these functions.

A previous functional analysis of the entire metagenome sampled from the bacterial community on the surface of *U. australis* [27], also obtained from the Sydney region, showed a different profile for the distribution of functional categories compared with those associated with gene cassettes observed in this study. For example, the proportion of proteins related to defence mechanisms in *U. australis* reported by Burke [27] was 10-fold lower than those associated with the gene cassettes observed here. This highlights that integrons do not sample genes randomly from the metagenome but are enriched with specific functions that provide cells with a selective advantage, such as enhanced defence capabilities.

A subset of 107 gene cassette ORFs were shared among all sample types across all sites (Fig. 4a). Of these, 92 gene cassettes (86 %) encoded entirely novel proteins with no homologues in the database. The remaining 15 gene cassettes (14 %) encoded proteins associated with functions such as virulence, antibiotic resistance, protein secretion, toxin/antitoxin systems, signal transduction, DNA modifications and cell membrane association (see Table S1, available in the online version of this article). Such functions are usually involved in interactions with other cells, whether in a positive or negative manner. Thus, the traits carried by these ubiquitous gene cassettes may render bacterial cells more competitive in interacting with other cells in environments such as densely packed biofilm communities.

### Cassette-encoded functions related to pathogenicity

**Fig. 6.**
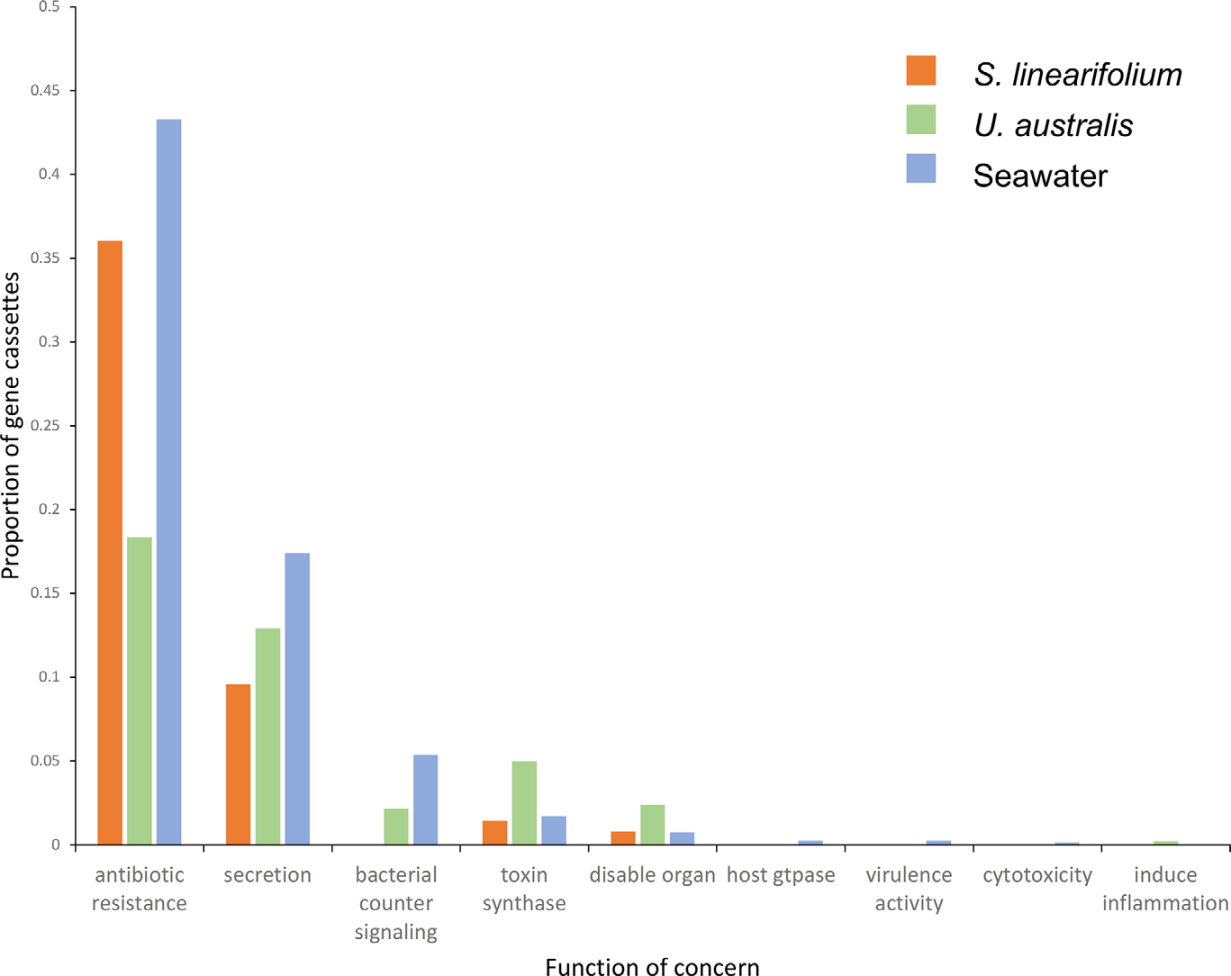
Proportion of gene cassette ORFs, for each sample type, classified under ‘functions of sequences of concern’ (SeqScreen).

Environmental biofilm communities may serve as reservoirs for pathogenicity traits, such as antibiotic resistance genes, which can represent a potential health hazard [28,29]. To provide a more detailed functional characterization of gene cassettes, ORFs obtained in all samples were screened using SeqScreen for potential pathogenicity traits [15]. Among the 220 158 gene cassette ORF sequences detected, 1161 (0.5 %) encoded proteins with functions related to pathogenicity. The great majority of these (~95 %) were classified under functional categories related to antibiotic resistance, secretion and toxin synthase, while the remaining gene cassettes (~5 %) included sequences related to bacterial counter-signalling, virulence activity, cytotoxicity and inflammation (Fig. 6). Detailed information on these functional categories is provided in Table S2.

ARGs were the most prominent category detected. Such ARGs are often carried by integron gene cassettes in clinical settings [30,31], and these were further analysed. Among the 722 ARGs detected, the majority were found in seawater and *S. linearifolium* samples, which contained approximately twice the number of ARG cassettes present in *U. australis* (Fig. 6). The majority of ARG cassettes encoded proteins that confer resistance to β-lactams, bleomycin and aminoglycoside antibiotics, which are the most common resistance types mediated by integrons [31,32]. While the primers used in this study do not recover information on the type of integron integrase gene adjacent to cassettes, such ARG cassettes are usually associated with mobile class 1 integron integrases [1,18]. The detection of ARG cassettes was not surprising, since the intertidal coastal locations from which the samples were collected are likely to experience high levels of anthropogenic pollution from water runoff. This can lead to pollution with commensal bacteria and with antimicrobial compounds, which would place a strong selective pressure on bacteria to acquire and disseminate ARGs via integrons.

### Predicted taxonomic origin of gene cassette based on *attC* sites

**Fig. 7.**
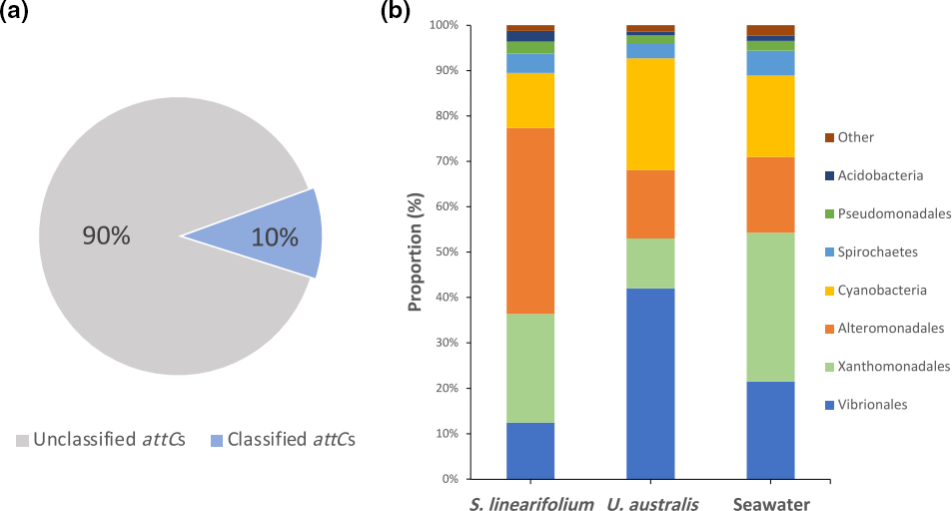
(**a**) Proportion of unclassified and classified gene cassette recombination sites (‘*attC*s’) based on models developed by Ghaly *et al*. [17]. (**b**) Proportion of the assigned taxa of origin of classified *attC*s among *S. linearifolium*, *U. australis* and seawater samples.

Out of the 138 593 *attC* sites detected among all samples, it was possible to identify the probable taxonomic source of 14 454 (~10 %) (Fig. 7a). This is lower than the 18.8 % of identified *attC* sites from gene cassettes recovered by Ghaly *et al*. [13] in a variety of soil and river sediment samples, suggesting that the models developed in this work may be less well suited to capture the variety of marine taxa involved in spreading integron gene cassettes in the marine environment. This is perhaps unsurprising as the marine environment has only rarely been the focus of integron/gene cassette surveys to date. In this study, most commonly identified *attC* sites were assigned to *Vibrionales, Xanthomonadales, Alteromonadales* and *Cyanobacteriales* (Fig. 7b), which are all bacterial taxa known to carry integrons [22,33]. These results agree with data that show the presence of these bacterial taxa in epiphytic biofilm communities associated with *U. australis* and *S. linearifolium* [(25, Unpublished data)].

## Conclusions

The present study, to our knowledge, represents the first to characterize the abundance, distribution and functions of integron gene cassettes in marine macroalgal-associated biofilms and surrounding seawater. In this work we show that the distribution of gene cassettes associated with epiphytic bacterial communities of *U. australis, S. linearifolium* and the surrounding seawater is driven by both the sample location and the sample type, with different macroalgae being host to significantly different sets of gene cassettes.

The findings from this work also strongly indicate that integrons are not sampling genes randomly from the surrounding gene pool but are enriched with gene cassettes that could give a selective advantage to the cell, enhancing its survival within the community. Interestingly, the distribution of functions carried by gene cassettes recovered from macroalga-associated biofilms was different between the bacterial communities of *U. australis* and *S. linearifolium*, indicating that the selection of gene cassettes could also depend on the specific traits of the host. For example, the COG functional category ‘Defence mechanisms’ was 6-fold more represented in *U. australis* epiphytic communities compared to *S. linearifolium* samples (Fig. 5b). We can speculate that the functions encoded by cassette genes in *U. australis* surface-associated biofilm cells might complement *Ulva*’s lack of chemical defenses. This further suggests that integrons might play an important role in the evolution of symbiotic relationships between bacterial communities and their macroalgal host. We also recovered a large number of integron gene cassettes carrying predicted ARGs, including ARGs that confer resistance to β-lactams, bleomycin and aminoglycoside antibiotics. This finding is perhaps not surprising given the anthropogenic impact and the broad selective advantage that ARG cassettes can offer.

While the full spectrum of functions carried by gene cassettes is likely to remain unknown in the near future, the integron gene cassette suites recovered here represent an important source of genetic novelty as well as harbouring sizable sets assigned to functional categories broadly associated with cell interactions. In addition, more than 10 % of these gene cassettes contained signal peptide domains that are associated with the cellular membrane or cellular export. These findings add to the growing evidence that many integron gene cassettes could encode proteins that have a role in facilitating cell-to-cell interactions and in shaping cell responses to changing environmental conditions. The results presented here pave the way for future studies into the roles integron gene cassettes play in contributing to the adaptive potential of bacteria within host-associated microbiomes, as well as providing better understanding of their distribution and diversity in aquatic environments.

## supplementary material

10.1099/mic.0.001446 Uncited Table S1.
