# Supplementary material for: Microbial biofilms on macroalgae harbour diverse integron gene cassettes
Source: Microbiology (Reading). 2024 Mar 15;170(3):001446. doi: 10.1099/mic.0.001446 (PMC10963911; doi:10.1099/mic.0.001446)
Supplement: Uncited Table S1. [file mic-170-01446-s001.pdf]

Supplementary Material

Table S1: Functional characterisation of the 15 gene cassettes shared among the microbial communities associated with *U. australis, S. linearifolium*, and seawater samples. The gene names, along with the putative organism of origin and the uniprot e-values are also reported.

Putative organism of origin	Gene name	Uniprot evalue
Paenibacillus antri	2''-aminoglycoside phosphotransferase	8.84E-08
Bacillus subtilis subsp. subtilis str. 168	Putative [ribosomal protein S5]-alanine N-acetyltransferase	1.28E-17
Escherichia coli O157:H7	Broad specificity amino-acid racemase YgeA	9.07E-86
Paenibacillus sp. FSL R7-0331	Signal transduction histidine-protein kinase/phosphatase DegS	2.9E-18
Chloroflexus aurantiacus J-10-fl	Probable RNA 2'-phosphotransferase	3.2E-70
Dichelobacter nodosus	Virulence-associated protein I	1.81E-21
Thermodesulfovibrio yellowstonii DSM 11347	Histidine kinase	7.12E-10
Coriobacterium glomerans PW2	Multifunctional fusion protein	2.27E-11
Calothrix sp. NIES-2100	Ribonuclease VapC	0.002
Proteus vulgaris	Endoribonuclease HigB	8.22E-27
Synechococcus elongatus PCC 7942 = FACHB-805	Synpcc7942_2319	2.8E-26
Variovorax paradoxus	Histidine kinase	3.25E-26
Martelella sp. AD-3	Signal peptidase I	2.04E-15
Congregibacter litoralis KT71	Abasic site processing protein	4.1E-39
Caulobacter vibrioides CB15	Toxin ParE1	3.9E-09

Table S2: Overview of the Functions of Sequences of Concern (FunSoCs), characterised by SeqScreen, that were detected in the ORFs of gene cassettes analysed in this study. Table was adapted from Balaji *et al.* (2022).

FunSoC Name	Category			FunSoC Definition	Protein Example	Citation
	V	B	E			
Disable organ	X	X	X	Disables an organ, but not necessarily by killing individual cells. This includes neurotoxins that block receptor conductance, superantigens, and toxins that degrade lung function, gut function, or are involved in the disruption of blood homeostasis.	Clostridium botulinum Botulinum neurotoxin type G (Q60393)	Rummel, A., et al.. (2007). Identification of the protein receptor binding site of botulinum neurotoxins B and G proves the double-receptor concept. Proceedings of the National Academy of Sciences, 104(1), 359-364.
Cytotoxicity	X	X	X	Kills cells by inhibiting a vital process such as translation, directly lysing the cells through pore formation, or destabilizing the plasma membrane	Escherichia coli Hemolysin E (Q68S89)	Oscarsson, J., et al. (1999). Molecular analysis of the cytolytic protein ClyA (SheA) from Escherichia coli. Molecular microbiology, 32(6), 1226-1238.
Induce inflammation	X	X	X	Directly activate host inflammatory pathways to cause damage	Bordetella pertussis Pertusis toxin S1 (P04977)	Connelly, C. E., et al. (2012). Pertussis toxin exacerbates and prolongs airway inflammatory responses during Bordetella pertussis infection. Infection and immunity, 80(12), 4317-4332.
Bacterial counter signalling		X		Bacterial suppression of host immune signalling within host cells to avoid inflammatory responses	Mycobacterium tuberculosis Protein EsxG (O53692)	Mehra, A., Zahra, A., Thompson, V., Sirisaengtaksin, N., Wells, A., Porto, M., ... & Philips, J. A. (2013). Mycobacterium tuberculosis type VII secreted effector EsxH targets host ESCRT to impair trafficking. PLoS pathogens, 9(10), e1003734.
Host gtpase		X		Target host small GTPases	Legionella pneumophila Phosphocholine transferase AnkX (Q5ZXN6)	Mukherjee, S., et al. (2011). Modulation of Rab GTPase function by a protein phosphocholine transferase. Nature, 477(7362), 103-106.
Virulence activity	X	X	X	Inclusive of a wide variety of virulence activities, including those that did not fit under another FunSoC category	Phytophthora infestans RxLR effector protein Avr3a (E2DWQ7)	Bos, J. I., et al. (2010). Phytophthora infestans effector AVR3a is essential for virulence and manipulates plant immunity by stabilizing host E3 ligase CMPG1. Proceedings of the National Academy of Sciences, 107(21), 9909-9914.
Antibiotic resistance		X	X	Counters the effect of antibiotics administered to inhibit the growth or vital functioning of bacterial or eukaryotic parasites. Note: These are not specific to pathogens.	Acinetobacter baumannii Beta-lactamase BlaGES-12 (C8ZII4)	By protein homology.
secretion		X		Bacterial secretion system components (T1SS - T8SS), including chaperones	Salmonella enteritidis Surface presentation of antigens protein SpaO (P0A1K8)	Li, J., et al. (1995). Relationship between evolutionary rate and cellular location among the Inv/Spa invasion proteins of Salmonella enterica. Proceedings of the National Academy of Sciences, 92(16), 7252-7256.
Toxin synthase		X	X	Enzymes that synthesize or modify toxins, particularly mycotoxins	Alternaria alternata Abhydrolase domain-containing protein (C9K1N0)	Miyamoto, Y., et al. (2008). Functional analysis of a multicopy host-selective ACT-toxin biosynthesis gene in the tangerine pathotype of Alternaria alternata using RNA silencing. Molecular plant-microbe interactions, 21(12), 1591-1599.
